# Treatment of Inflammatory Bowel Disease: A Comprehensive Review

**DOI:** 10.3389/fmed.2021.765474

**Published:** 2021-12-20

**Authors:** Zhaobei Cai, Shu Wang, Jiannan Li

**Affiliations:** ^1^Department of General Surgery, The Second Hospital of Jilin University, Changchun, China; ^2^Department of Gastroenterology and Hepatology, Chinese People's Liberation Army General Hospital, Beijing, China; ^3^Department of Radiotherapy, The Second Hospital of Jilin University, Changchun, China

**Keywords:** inflammatory bowel disease, Crohn disease, ulcerative colitis, therapeutics, recent advance

## Abstract

Inflammatory bowel disease (IBD), as a global disease, has attracted much research interest. Constant research has led to a better understanding of the disease condition and further promoted its management. We here reviewed the conventional and the novel drugs and therapies, as well as the potential ones, which have shown promise in preclinical studies and are likely to be effective future therapies. The conventional treatments aim at controlling symptoms through pharmacotherapy, including aminosalicylates, corticosteroids, immunomodulators, and biologics, with other general measures and/or surgical resection if necessary. However, a considerable fraction of patients do not respond to available treatments or lose response, which calls for new therapeutic strategies. Diverse therapeutic options are emerging, involving small molecules, apheresis therapy, improved intestinal microecology, cell therapy, and exosome therapy. In addition, patient education partly upgrades the efficacy of IBD treatment. Recent advances in the management of IBD have led to a paradigm shift in the treatment goals, from targeting symptom-free daily life to shooting for mucosal healing. In this review, the latest progress in IBD treatment is summarized to understand the advantages, pitfalls, and research prospects of different drugs and therapies and to provide a basis for the clinical decision and further research of IBD.

## Introduction

Inflammatory bowel disease (IBD), including ulcerative colitis (UC) and Crohn's disease (CD), is a chronic and recurrent inflammatory disease that mainly relates to the intestinal tract. Over recent decades, the epidemiology of IBD has changed considerably. The early twenty-first century has witnessed a rapidly rising incidence in newly industrialized countries (1). The morbidity of IBD was about 1.74 per 100,000 person-years in China (2). Although the morbidity turns to be stable in western countries, burden remains high as prevalence exceeds 0.3% (1). As a global disease, IBD not only seriously endangers human health but also brings heavy financial burdens to individuals, families, and society.

The exact cause of IBD remains indistinct, but it is generally accepted that its etiopathology is multifactorial, involving genetic predisposition, mucosal barrier dysfunction, disturbances in the gastrointestinal microbiota, dysregulated immune responses, environmental, and lifestyle factors (3, 4).

The past few years have seen an expansion in IBD therapeutic options. Conventional treatments control symptoms through pharmacotherapy, including aminosalicylates, corticosteroids (CSs), immunomodulators, and biologics, with other general measures and/or surgical resection if necessary. The introduction of specific inhibitors of tumor necrosis factor (TNF) is a groundbreaking achievement, enabling long-standing remission, and modification of the IBD course in a significant fraction of patients (5). However, primary non-response to TNF inhibitors was observed in up to 40% of patients in clinical trials and 10–20% patients in clinical series; secondary loss of response occurred in ~23–46% of patients after 1 year of treatment (6), which calls for new therapeutic strategies. New therapeutic strategies are emerging, involving small molecules, apheresis therapy, improved intestinal microecology, cell therapy, and exosome therapy. In addition, patient education on diet and psychology appears to benefit IBD treatment.

Recent progress in therapeutic approaches, especially the emergence of biologics, has not only promoted the transformation of the treatment mode in IBD, but also changed the perspective of IBD therapy. Traditionally, the therapeutic effects are mainly evaluated through clinical symptom score. Nowadays, the disease activity can also be assessed by objective indicators such as endoscopic findings and biomarkers (7). The goals are not only to induce and maintain remission in symptom, to prevent and treat complications but also to achieve mucosal healing. Mucosal healing refers to the elimination of local mucosal inflammation and the restoration of the normal mucosal structure. Although there is still no unified criteria for the determination of mucosal healing, it is usually characterized by the disappearance of endoscopic ulcer (8). Multiple studies are emerging to show that mucosal healing may be associated with reduced rates of clinical recurrence, hospitalization, surgery and disability, and a good long-term prognosis (9–11).

In this review, we not only focus on drugs and therapies that have been approved, but also focus on the potential methods for the treatment of IBD, providing a comprehensive overview for clinicians of available therapies and drugs for IBD treatment.

From the database's inception until October 2021, we conducted a comprehensive search in PubMed and Web of Science. The retrieval strategy is based on Medical Subject Headings (MeSH) and corresponding free words. The major search terms are as follows: “Inflammatory Bowel Disease,” “Bowel Diseases, Inflammatory,” “IBD,” “Crohn's Disease,” “Crohn's Enteritis,” “Ulcerative Colitis,” “Colitis Gravis,” “Aminosalicylates,” “Mesalazine,” “5-Aminosalicylic Acid,” “Corticosteroids,” “Thiopurines,” “Methotrexate,” “Calcineurin Inhibitors,” “Biologics,” “Janus Kinase inhibitors,” “Ozanimod,” “Etrasimod,” “Surgical Procedure,” “Apheresis,” “Blood Component Removals,” “Antibiotics,” “Antibacterial Agents,” “Probiotics,” “Prebiotics,” “Synbiotics,” “Postbiotics,” “Fecal Microbiota Transplantation,” “Stem Cell Transplantations,” “Exosomes,” “Diet.” The above search terms were connected by the logical operators “OR” or “And.” The research focused on the treatment of IBD. A total of 9,885 references were retrieved. Studies, which are old, repetitive and non-English and those without clear information were excluded. We selected some representative scientific papers and 257 references were finally quoted.

## Pharmacological Intervention

At present, pharmacological intervention is important for IBD treatment. The medications mainly include aminosalicylates, CSs, immunomodulators, biologics, and oral small molecules. We mainly introduced their mechanism of action, efficacy, and safety in UC or CD.

### Aminosalicylates

Aminosalicylates for IBD mainly include traditional sulfasalazine (SASP) and other types of 5-aminosalicylic acid (5-ASA) drugs. SASP is composed of 5-ASA and sulphapyridine (SP) by diazo bonding and has been used to treat IBD for 80 years. In the treatment of IBD, SASP is the prodrug, SP is the carrier, and 5-ASA is the active part. The mechanisms of action (MOA) of 5-ASA and SASP include interference with the metabolism of arachidonic acid (conversion to prostaglandin and leukin-triene), scavenging of reactive oxygen species, and effects on the function of white blood cells and the production of cytokines (12). Oh-oka et al. have proposed a novel anti-inflammatory mechanism in the colitis treatment: 5-ASA could induce regulatory T cells (Tregs) in the colon through the aryl hydrocarbon receptor pathway, followed by the activation of transforming growth factor (TGF)-β (13).

Studies on the efficacy and safety of 5-ASA preparations in the treatment of IBD are summarized in Table 1. Recent studies have reported that oral 5-ASA has better efficacy in UC treatment than placebo and shows similar effects (clinical remission rates) between once-daily dosing and conventional (twice or three times daily) dosing (14). The efficacy of SASP in UC treatment is similar to that of other 5-ASA preparations. However, taking costs into account, SASP may be the preferred option in clinical application, because other 5-ASA preparations are more expensive (14). A case-control study has reported that 5-ASA maintenance therapy can reduce the risk of colorectal cancer by 75% in UC patients (15). A meta-analysis has proved the efficacy of topical 5-ASA in preventing relapse of UC (16).

**Table 1:** Aminosalicylates.

**Type of study**	**Patients**	**Treatment**	**Therapy period**	**Results/Conclusion**	**References**
A review	UC patients	Oral 5-ASA	NA	5-ASA was more effective than placebo. There was no difference in clinical remission rates between once-daily dosing and conventional (twice or three times daily) dosing. Other 5-ASA formulations appeared to be as efficacious as SASP	(14)
A case-control study	UC patients	5-ASA	NA	Regular 5-ASA therapy reduced colorectal cancer risk by 75%	(15)
A meta-analysis	Patients with quiescent UC	5-ASA	6–24 months	Topical 5-ASA was effective in preventing relapse of UC in remission	(16)
A systematic review	CD patients	Oral 5-ASA	NA	No significant advantage was found in oral 5-ASA for the maintenance of medically-induced remission	(17)
A retrospective study	Adults with CD	5-ASA	NA	5-ASA was widely used as a long-term treatment for CD. The use of CD-related healthcare resources decreased significantly in the year following 5-ASA initiation	(18)
An updated cochrane review	CD patients in remission after surgery	Oral 5-ASA	NA	5-ASA drugs were superior to placebo for maintaining surgically-induced remission of CD. 5-ASA formulations appeared to be safe when compared with placebo, no treatment or biologics	(19)
A bayesian network meta-analysis	Mild-to-Moderate CD patients	Mesalamine, SASP, CSs, and budesonide	8–17 weeks	CSs and high-dose budesonide were effective treatments for inducing remission in mild-to-moderate CD. CSs were more effective than high-dose mesalamine, but high-dose mesalamine was an option among patients preferring to avoid steroids	(20)
A systematic review and meta-analysis	Adults with luminal CD in remission after a surgical resection	5-ASA	NA	5-ASA was of modest benefit in preventing relapse of quiescent CD after a surgical resection	(21)
A systematic review	Patients with mildly to moderately active CD	Aminosalicylates	NA	For induction therapy of mild to moderate CD, SASP had modest efficacy and high dose mesalamine (3–4.5 g/day) was not superior to placebo.	(22)

*UC, ulcerative colitis; 5-ASA, 5-aminosalicylic acid; NA, not applicable; SASP, sulfasalazine; CD, Crohn's disease; CSs, corticosteroids*.

The therapeutic efficacy of aminosalicylic acid preparations for CD remains controversial. A review has suggested that oral 5-ASA preparations have no significant advantage in maintaining remission in patients with CD (17). However, a retrospective study in the UK found that 5-ASA was widely used as a long-term treatment for CD as about a quarter of patients continued to use 5-ASA for more than 10 years (18). 5-ASA therapy for more than a year could reduce the consumption of related medical resources (including referrals, hospitalization, and surgery) (18). Gjuladin-Hellon et al. have reported the benefit of 5-ASA in preventing relapse of CD in remission after surgery (19). Coward et al. in their Bayesian network meta-analysis found that high-dose mesalamine is an option for inducing remission among mild-to-moderate CD patients preferring to avoid steroids (20). Other studies have also reported the treatment effectiveness of aminosalicylates in CD (21, 22).

Side effects associated with 5-ASA, including flatulence, nausea, abdominal pain, diarrhea, and headache, are generally mild. In contrast, the side effects of SASP, such as infertility, hemolytic anemia, photosensitization, and granulocytosis, are much more than those of 5-ASA (12). However, a few patients may develop nephrotoxicity within 1 year of 5-ASA administration (23).

### CSs

Oral CSs have been used for IBD treatment since the 1950s (24, 25), and can effectively induce remission when a flare occurs. CSs combine with CSs receptors in the cytoplasm, and then CSs receptors are activated. The activated CSs receptors could get into the nucleus and interact with specific proinflammatory transcription factors (such as nuclear factor-kappaB and activator protein-1), which will recruit co-activator complexes (for e.g., histone deacetylation enzymes) to inhibit the transcription of some inflammatory genes (26). Moreover, activated CSs receptors can also bind to specific response elements in the promoter region of anti-inflammatory genes in the nucleus to regulate the expression of anti-inflammatory genes. In addition, the anti-inflammatory effect of CSs may be mediated by different membrane receptors (27).

CSs may be a kind of treatment selection for patients with UC who have not responded to mesalazine within 2–4 weeks, and those with mild-to-moderate CD, especially with extensive lesions (28). CSs have no proven efficacy in maintaining remission in IBD and should not be used for this purpose. Systemic oral CSs may result in numerous side effects, such as opportunistic infections, diabetes mellitus, hypertension, ocular effects, venous thromboembolism (VTE), osteoporosis, etc (29, 30). Steroid dependency or excess was found in ~15–40% of IBD patients (31, 32). Further investigation should define appropriate corticosteroid use and find measures for the improvement in CSs prescription management.

Studies related to CSs' efficacy in IBD are presented in Table 2. Systematic reviews and metanalyses have proved the benefits of CSs in inducing remission of IBD (33). A recent study demonstrated that CSs were more effective than 5-ASA in the treatment of CD (20). Other studies have also reported the efficacy and safety of CSs in IBD treatment (34–40).

**Table 2:** Corticosteroids.

**Type of study**	**Patients**	**Treatment**	**Results/Conclusion**	**Adverse events**	**References**
A review	IBD patients	CSs and aminosalicylates	There were numerous adverse events of CSs, particularly at high doses and prolonged treatment. Therapy with budesonide may result in a better safety profile. 5-ASA treatment is usually well-tolerated, but with regard to the rare nephrotoxic events	CSs: opportunistic infections, diabetes mellitus, hypertension, ocular effects (glaucoma and cataracts), psychiatric complications, hypothalamic-pituitary-adrenal axis suppression and increased fracture risk	(29)
A systematic review	UC patients	Second-Generation oral CSs	Beclomethasone dipropionate and budesonide MMX have better efficacy in the induction of remission in UC than placebo or mesalazine. Second-generation CSs have a more favorable safety and tolerability than systemic CSs	Altered glucose concentration, constipation, menorrhagia, UC exacerbation, headache, nausea	(30)
A multi-center audit	IBD patients	CSs	14.9% of British patients with IBD experienced steroid dependency or excess	NA	(31)
A systematic review and meta-analysis	IBD patients	CSs	CSs were beneficial for inducing remission in UC, and might be effective in CD. Standard CSs were more effective than budesonide	NA	(33)
A prospective observational study	Adult outpatients with UC or CD	Oral prednisone (40 mg/day for 2 weeks, followed by a tapering course of 5 mg/day reduction every week)	CSs was associated with high rate of mood change in IBD patients when disease flares	Frequentmood changes	(34)
A systematic review and meta-analysis	IBD patients with CMV	CSs, TPs, TNF antagonists	Exposure to CSs or TPs, but not anti-TNF drugs, was associated with an increased risk of CMV reactivation in IBD patients	CMV reactivation	(35)
A retrospective review	IBD patients	CSs	Prolonged use of CSs was associated with significant harm to IBD patients	VTE, fragility fracture, infections	(36)
A retrospective survey	UC patients	Oral or intravenous CSs	The majority of UC patients primarily responded to CSs. But after 1 year of treatment, nearly half of patients were assessed as CS dependence	NA	(37)
A retrospective study	Adults with IBD	CSs	The use of CSs significantly increased the risk of VTE	VTE	(38)
A population-based cohort study with a nested case-control analysis	Incident IBD patients aged ≥66 years	Systemic oral CSs	Oral CSs were associated the increase risk of serious infections in elderly-onset IBD patients	Diabetes, chronic respiratory diseases, chronic kidney diseases, cancer	(39)
A retrospective cohort study	UC patients	CSs	About half of newly-diagnosed patients with UC required CSs. Among CS users, one third of the patients had a sustained response after the initial CSs course while two-thirds required further CSs therapy	NA	(40)
Two randomized, double-blind, placebo-controlled, phase 3 studies	Patients with mild-to-moderate active UC	Budesonide MMX (9 or 6 mg once daily)	Budesonide MMX 9 mg resulted in significantly higher combined clinical and colonoscopic remission rates (*P* = 0.0002)	Headache, nausea, abdominal pain, nasopharyngitis	(41)
A phase III, randomized, double-blind, double-dummy, placebo-controlled, parallel-group trial	Patients with active, mild-to-moderate UC	Budesonide MMX (9 mg/day)	Budesonide MMX 9 mg appeared to be safe and more effective than placebo at inducing combined clinical and endoscopic remission in patients with active, mild-to-moderate UC	Headache, flatulence, nausea, blood cortisol decrease	(42)

*IBD, inflammatory bowel disease; CSs, corticosteroids; 5-ASA, 5-aminosalicylic acid; UC, ulcerative colitis; MMX, Multi Matrix; NA, not applicable; CD, Crohn's disease; CMV, cytomegalovirus; TNF, tumor necrosis factor; VTE, venous thromboembolism*.

Second-generation oral CSs, such as budesonide, are becoming available and may have better safety and tolerability profile than conventional CSs. Target delivery of steroids to the site of inflammation potentially reduces systemic side effects (30). Budesonide is a synthetic CS with a high affinity for CSs receptors. While pH-dependent budesonide capsules restrict the release of budesonide to the distal ileum and ascending colon, budesonide Multi Matrix (MMX) is released throughout the entire colon. The tolerability of budesonide MMX at 8 weeks was similar to that of placebo, mesalazine (41), and pH-dependent budesonide (42), which may prompt the use of budesonide in patients who are not suitable for conventional CSs. For the mild-to-moderate CD of the ileum and/or ascending colon, 9 mg budesonide once daily for 8 weeks is recommended with a 2 week taper (28).

### Immunomodulators

Immunomodulators are important for patients with IBD and mainly include thiopurines (TPs), methotrexate (MTX), calcineurin inhibitors, and Janus Kinase (JAK) inhibitors. The studies on the efficacy and safety of immunomodulators in IBD are summarized in Supplementary Table 1.

#### TPs

During the progression of IBD, activated T lymphocytes infiltrate the inflammatory site of the intestinal mucosa and produce a variety of cytokines, further aggravating intestinal inflammation. TPs, including azathioprine (AZA), 6-mercaptopurine (MP), and 6-thioguanine (TG) could control intestinal inflammation by inhibiting T lymphocyte proliferation and activation. These inactive 6-TP prodrugs are metabolized into pharmacologically active deoxy-6-thioguanosine phosphate (deoxy-6-TGNP). Deoxy-6-TGNP can interfere with DNA synthesis and inhibit lymphocyte proliferation. Besides, 6-TGNP can bind to Rac1 to form the 6-TGNP-Rac1 complex, thus blocking the activation of Rac1 in T lymphocytes and inhibiting the survival and function of T lymphocytes (43).

It has been proved that AZA has a favorable and similar therapeutic effect on CD and UC, which helps reduce hospitalization and surgery rates of IBD patients (44–46). A meta-analysis has indicated that AZA/6-MP is more effective in preventing UC recurrence than placebo (OR = 2.59, 95% CI: 1.26–5.3) (47). A retrospective cohort study has shown TP's long-term efficacy on UC patients, with a 7-year maintenance remission rate of 43.9% and a colectomy-free survival rate of 88% (48). A prospective, observational study has reported that 70% of steroid-dependent CD patients treated with AZA achieved a 60 month steroid-free remission (49).

However, TPs have many adverse side effects, such as bone marrow suppression (50), liver injury (51), and gastrointestinal intolerance (44), etc. It has been reported that up to 39% of patients with IBD discontinue using TPs due to adverse reactions, most of which occur within 3 months of treatment (44). The use of TPs in IBD treatment has declined due to concerns about adverse drug reactions.

#### MTX

Low doses of MTX can inhibit the function of several enzymes related to DNA synthesis, and downregulate a variety of inflammatory cytokines [such as interleukin (IL)-1, IL-2, IL-6, IL-8, etc.], thus inhibiting the proliferation of T lymphocytes and inflammatory response (52). A study has found that 72% of patients with active CD achieved clinical remission after 3 months of MTX treatment (53). A randomized controlled trial has shown that in patients with CD who have clinical remission after intramuscular treatment with 25 mg MTX per week, the rate of maintenance remission at 40 weeks of intramuscular treatment with 15 mg MTX per week is 65%, higher than that of the control group (39%, *P* = 0.04) (54). MTX has not been proven to have efficacy in inducing remission in UC (55). In addition, MTX can cause fatigue, nausea, vomiting, diarrhea, peritoneal abscess, hypoalbuminemia, atypical pneumonia, severe rash, etc. (27, 55).

#### Calcineurin Inhibitors

Calcineurin, activated by calmodulin, can induce dephosphorylation and activation of the nuclear factor of activated T cells (NFAT). Then the activated NFAT will move from cytoplasm to nucleus and combine with the gene regulatory regions to regulate gene transcription of a variety of inflammatory cytokines [TNF-α, interferon (IFN)-c, IL-2, etc.] (56). Calcineurin inhibitors, including Cyclosporine A (CsA) and Tacrolimus (TAC) interfere with the signaling pathway, and therefore inhibit the inflammatory response. CsA and TAC bind to intracellular Cyclophilin A and FK binding protein 12, respectively, forming complexes that inhibit NFAT dephosphorylation (56). In addition, it has been reported that TAC not only has an immunosuppressive effect on T cells, but also inhibits the activation and promotes the apoptosis of macrophages, thereby inhibiting the production of pro-inflammatory cytokines (IL-12/IL-23 and TNF-α) (57).

A randomized controlled trial demonstrated that more than 80% of patients with severe acute refractory UC responded to CsA (58). It has been reported that the 8-day clinical remission rate (84.2 vs. 85.7%) of patients with severe UC treated with intravenous 4 mg/kg CsA is similar to that of patients treated with intravenous 2 mg/kg CsA (59). Stange et al. performed a randomized controlled trial to delineate the long-term effect of CsA on chronic active CD and found that CsA combined with low-dose steroids had no advantage over the sole use of low-dose steroids (60).

Although the MOA of TAC is similar to that of CsA, the immunosuppressive effect of TAC is much higher than that of CsA and is 10–20 times *in vivo* and 30–100 times *in vitro*, respectively (61). Additionally, TAC is well-absorbable through the intestine, and it is similarly effective for refractory UC whether administered intravenously or orally (62). Multiple studies have confirmed the effectiveness of TAC in patients with refractory UC (63–67). A randomized controlled trial showed that 68.4% of patients with refractory UC taking TAC orally after 2 weeks had an improved disease activity index (DAI) score (>4 points, all categories improved), but in the control group, only 10% of patients' score was improved (*P* < 0.001) (63). Yamamoto et al. have revealed that 77.8% of patients with refractory UC respond to TAC and 70.4% of patients have clinical remission within 30 days (64). Komaki et al. performed a systematic review and meta-analysis to exam the efficacy of TAC as rescue therapy for active UC and found that the 2-week clinical response rate of TAC was significantly higher than that of placebo (RR = 4.61, 95% CI: 2.09–10.17) (67). The clinical response rates at 1 and 3 months were 73% (95% CI: 64–81%) and 76% (95% CI: 59–87%), and the colectomy-free rates at 1, 3, 6, and 12 months were 86, 84, 78, and 69%, respectively. However, the efficacy of TAC in CD treatment remains controversial. McSharry et al. have systematically reviewed the studies assessing the potency of TAC in the treatment of luminal CD and found that the roughly computed remission rate was 44.3% (7–69%) and the partial response rate was 37.1% (14–57%) (68). Iida et al. have probed into the studies from 1950 to December 2017 to show the efficacy of TAC in CD treatment and found that the clinical remission rates for luminal CD patients systemically treated with TAC, perianal CD patients with systemic TAC treatment, and localized CD patients with topical administration of TAC were 37.1, 32.0, and 22.7%, respectively (69).

TAC has a high incidence of adverse side effects, including tremor, renal function damage, infectious diseases, hot flashes, hyperkalemia, headache, etc (63, 64), which should be taken into consideration during clinical practice. Besides, the blood concentration and the patient's general status should be closely monitored when using TAC. The optimal blood trough concentration appeared to be 10–15 ng/ml during remission-induction therapy for refractory UC (63). Recently, a study has reported that the use of calcineurin inhibitors makes 1-year colectomy rates of UC patients who are previously exposed to biologics significantly higher than those of patients who are biologic-naïve (70). Clinical studies exploring the efficacy and safety of CsA and TAC in the treatment of IBD are rare, and more randomized controlled trials are needed.

### Biologics

Biologics mainly include pro-inflammatory cytokine inhibitors and integrin antagonists. The pro-inflammatory cytokines, TNF-α and IL-12/23, play an important role in the pathogenesis of IBD. Studies on the efficacy and safety of biologics in IBD treatment are summarized in Supplementary Table 2.

#### Anti-TNF Therapy

TNF-α is a prototypic member of a large family of cytokines that play important roles in inflammation, apoptosis, proliferation, invasion, etc. (71) Overexpression of TNF-α can cause chronic inflammation and lead to autoimmune diseases and tissue damage. Anti-TNF-α monoclonal antibodies, such as Infliximab (IFX) and Adalimumab (ADA), exert therapeutic effects by inhibiting TNF-α-associated inflammatory responses and tissue damage.

IFX therapy may be applied for the treatment of patients who are intolerant or do not respond well to CSs and immunomodulators and are steroid-dependent. A randomized controlled trial evaluating the efficacy of IFX in patients with moderate to severe UC demonstrated that colorectomy rates decreased by 7% after 54 weeks of IFX treatment. UC-related hospitalization and surgery rates saw a decrease as well (72). Present et al. found that the clinical response rate of patients with CD was 68% after intravenous injection of 5 mg/kg IFX, and the complete healing rate of the fistula was 55% (73). The efficacy of IFX was shown in about 2 weeks, and the median time of fistula closure was 3 months. Golimumab is a fully human IgG1 monoclonal antibody against TNF-α with good efficacy and safety. It is approved for use in patients with moderate to severe UC (74, 75) and CD (76, 77), who fail to respond to conventional therapy.

Anti-TNF treatment is not all-encompassing despite its vital role in IBD treatment. Up to 40% of patients do not respond to TNF inhibitors, and nearly 23–46% of patients experience secondary loss-of-response 1 year after anti-TNF-α treatment (6). It may be possible to achieve long-term remission through dose escalation, shorter intervals between infusions (78) or combination therapy (79). Due to anti-TNF agents' dose-related therapeutic benefit, measurement of serum trough level and anti-drug antibody is advocated (80, 81).

#### Anti-IL-12/23 Therapy

IL-12 and IL-23 are important pro-inflammatory cytokines in intestinal inflammation, mainly produced by antigen-presenting cells. IL-12 is composed of the p35 and p40 subunits, and IL-23 is composed of the p40 and p19 subunits. Preclinical studies have suggested that IL-12 and IL-23 are involved in the pathophysiological process of IBD and play a role in the induction and maintenance of intestinal inflammation (82). In addition, genomic studies have shown an association between the IL-12/IL-23 pathway and CD (83).

Ustekinumab is a fully humanized IgG1 monoclonal antibody that binds to the common p40 subunit of IL-12 and IL-23 to inhibit the binding of IL-12 and IL-23 to the IL-12 receptor on the cell membrane surface of T cells and NK cells, thereby inhibiting intestinal inflammation. Ustekinumab has been approved be effective for the treatment of moderate to severe CD and UC (84). Rutgeerts et al. conducted three-phase randomized controlled clinical trials and found that the 8-week simplified endoscopic activity score for CD decreased by 2.8 in the ustekinumab group while the decrease in the control group was only 0.7 (*P* = 0.012), showing that ustekinumab had a better treatment efficacy than placebo in patients with CD (85). Feagan et al. reported that for patients with moderate to severe CD, the clinical response rates at week 6 in the intravenous ustekinumab group were significantly higher than those in the control group, and the clinical remission rates at week 44 of patients receiving intravenous ustekinumab were also higher than those of patients receiving placebo (86).

Mirikizumab is a humanized monoclonal antibody that targets the unique p19 subunit of IL-23. In a phase 2 trial, patients with moderate to severe UC were randomly divided into 4 groups and were given intravenous placebo, 50 mg mirikizumab, 200 mg mirikizumab, and 600 mg mirikizumab, respectively (87). It was reported that clinical remission rates of patients given 200 mg mirikizumab at week 12 were significantly higher than those of patients given placebo. The clinical remission rates of the other two mirikizumab groups were not significantly different from those of the placebo group. No serious adverse events or unexpected safety problems occurred in any groups during the induction and maintenance treatment.

Risankizumab, a humanized IgG monoclonal antibody against the p19 subunit of IL-23, undergoes phase 2 and phase 3 clinical evaluation. A randomized, double-blind, placebo-controlled phase 2 study demonstrated that risankizumab was superior to placebo in inducing clinical remission in patients with moderate to severe CD (88). Feagan et al. conducted an open-label extension study in patients with moderate to severe CD. It was proved that extended intravenous induction with risankizumab effectively increased clinical response and remission rates at week 26 and subcutaneous maintenance therapy with risankizumab achieved sustained remission until week 52 in ~70% of patients were in clinical remission at week 26 (89).

Multiple studies have suggested that IL-12/23 and IL-23 antagonists are potential therapeutic options for IBD treatment. Experts recommended IL-12/23 and IL-23 antagonists as a first- or second-line therapy because of their efficacy in biologic-naïve and experienced patients (90).

#### Anti-integrin Therapy

Integrin, a cell surface glycoprotein receptor, mediates the homing of leucocytes into surrounding tissues by binding to tissue-specific cell adhesion molecules (CAMs). α4β7 integrin plays a key role in the homing of leucocytes to the intestinal mucosa and related lymphoid tissues. The homing of intestinal selective leucocytes is mediated by the binding of α4β7 integrin and mucosal addressin cell adhesion molecule (MAdCAM)-1 (91). The degree of α4β7+ cell infiltration and MAdCAM-1 expression are increased in the intestinal tracts of IBD patients.

Additionally, the specific binding of αEβ7 integrin on leukocytes to E-cadherin on epithelial cells (especially intestinal epithelial mucosal cells) is thought to mediate cell retention (92). The accumulation of leukocytes in the intestinal tract aggravates intestinal inflammatory response. Under this condition, T lymphocytes produce more pro-inflammatory cytokines (IFN-γ, TNF-α, and IL-17A) and IL-9, a cytokine that inhibits epithelial cell repair. These produced cytokines are considered to be important in the pathogenesis of IBD (93). Anti-integrin therapy blocks the effect of integrin on the surface of leucocytes and endothelial CAMs, thereby inhibiting leukocytes from interacting with the intestinal mucosa.

Vedolizumab, a recombinant human IgG1 monoclonal antibody, specifically inhibits the binding of α4β7 integrin to MAdCAM-1, preventing lymphocyte migration to the intestinal tissue, and thereby alleviating local intestinal inflammation. The three-phase randomized controlled clinical trials have proved the effectiveness of vedolizumab in inducing and maintaining remission in patients with IBD (94, 95). In a comprehensive study, vedolizumab has shown great tolerability and safety (96), which may be due to that the intestinal selectivity help to avoid adverse effects of systemic immunosuppression. Based on these findings, vedolizumab has been approved in the United States and Europe to be applied for adult patients with moderate to severe UC and CD showing no response or tolerance to conventional therapy or anti-TNF-α monoclonal antibodies (97). Vedolizumab can only block lymphocyte migration to the intestinal tract and does not directly control the mucosal inflammatory response. Vedolizumab in combination therapy with calcineurin or TNF-α inhibitors is another choice for patients with refractory IBD (98, 99).

Etrolizumab is a monoclonal antibody that selectively targets the β7 subunit of both α4β7 and αEβ7 integrins. Its gut-selectivity and the dual mechanism of action make it an alternative option for IBD treatment (93). A randomized, double-blind, placebo-controlled, phase 2 study indicated that etrolizumab was more effective than placebo in inducing clinical remission at week 10 for patients with moderate-to-severe UC (100). Phase 3 clinical trials are ongoing and several of them have demonstrated that etrolizumab is more effective than placebo in inducing remission for patients with moderate to severe UC (101). The efficacy of etrolizumab in maintaining remission remains to be confirmed. No major safety issues have been found to date.

Carotegrast Methyl (AJM300) is an orally active small molecule inhibitor that specifically targets the α4 subunit of α4β7 and α4β1. A double-blind, placebo-controlled, phase 2a study demonstrated that AJM300 was more effective than placebo at week 8 for patients with UC and had an acceptable safety profile (102).

Additionally, PF-00547659, a fully human monoclonal antibody targeting MAdCAM-1, has been proved to be well-tolerated and effective in inducing remission in patients with moderate to severe UC (103). PN-943 is another emerging orally administered α4β7 antagonist peptide and it has been confirmed that PN-943 is effective for the induction of remission in UC (104). A phase 2 study is ongoing to evaluate the effects of PN-943 (150 and 450 mg twice daily) on moderate to severe UC patients (NCT04504383).

Biological agents are expensive despite the advantages of high selectivity, high efficiency and low toxicity. Besides, primary no-response, secondary loss-of-response, and therapeutic intolerance in IBD treatment with biologics urge researchers to actively explore other therapies.

### Small Molecules

Orally absorbed small molecules have attracted great interest of researchers because of the convenience of oral administration. Studies on the efficacy and safety of small molecules for IBD are listed in Table 3.

**Table 3:** Small molecules.

**Type of study**	**Patients**	**Treatment**	**Median treatment duration**	**Median follow-up duration**	**Results/Conclusion**	**Adverse events**	**References**
Three phase 3, randomized, double-blind, placebo-controlled trials	Adults with UC	Tofacitinib (induction therapy:10 mg twice daily for 8 weeks; maintenance therapy: either 5 or 10 mg twice daily for 52 weeks)	8, 8, 52 weeks	8, 8, 52 weeks	Tofacitinib appeared more effective in inducing and maintaining remission in patients with active CD compared with placebo	Increased lipid levels, infections, cardiovascular events	(105)
A phase 2, double-blind, randomized, placebo-controlled trial	Patients with moderate-to-severe CD	Filgotinib (GLPG0634, GS-6034) (200 mg once daily)	10 weeks	20 weeks	Filgotinib was more effective for inducing remission than placebo, and it had an acceptable safety profile	Infections	(106)
A multicenter, double-blind, phase 2b study	Adults with moderately to severely active UC and an inadequate response, loss of response, or intolerance to CSs, immunosuppressors, and/or biologics	Upadacitinib (7.5, 15, 30, or 45 mg once daily)	8 weeks	8 weeks	Upadacitinib (45 mg) was more efficacious as induction therapy than placebo	Increased serum lipid levels and creatine phosphokinase, herpes zoster, pulmonary embolism, deep venous thrombosis	(107)
A double-blind, placebo-controlled phase 2 trial	Adults with moderate-to-severe UC	Ozanimod (RPC1063) (0.5 or 1 mg daily)	32 weeks	32 weeks	Ozanimod at a daily dose of 1 mg resulted in a slightly higher rate of clinical remission of UC than placebo	Pyrexia, arthralgia, alanine aminotransferase increased, rash, vomiting, orthostatic hypotension, aspartate aminotransferase increased, hyperbilirubinemia, insomnia, nasopharyngitis, proctalgia	(108)
A phase 3, multicenter, randomized, double-blind, placebo-controlled trial	Patients with moderately to severely active	Oral ozanimod hydrochlorid (1 mg once daily) for induction therapy	10, 10, 52 weeks	10, 10, 52 weeks	Ozanimod resulted in significantly increased incidences of clinical response and clinical remission for both induction and maintenance period	Elevated liver aminotransferase levels, nasopharyngitis, headache, arthralgia	(109)
A single-arm, phase 2, prospective observer-blinded endpoint study	Adults with moderately to severely active CD	Ozanimod (0.25 mg daily for 4 days, followed by 3 days at 0.5 mg daily, then 1.0 mg daily for a further 11 weeks, followed by a 100-week extension)	12 weeks	112 weeks	Endoscopic, histological, and clinical improvements were seen within 12 weeks of initiating ozanimod therapy in patients with moderately to severely active CD	CD(flare), abdominal pain, lymphopenia, arthralgia, nausea	(110)
A phase 2, proof of concept, double-blind, parallel-group study	Patients with moderately to severely active UC	Etrasimod (APD334) (1 or 2 mg once daily)	12 weeks	12 weeks	Erasimod 2 mg was more effective than placebo in producing clinical and endoscopic improvements	Anemia, urinary tract infection, headache, blood creatine phosphokinase increased, gamma-glutamyltransferase increased, sinusitis, fever, hyperlipasemia	(111)

*UC, ulcerative colitis; CD, Crohn's disease; CSs, corticosteroids*.

#### JAK Inhibitors

As novel therapeutic drugs, JAK inhibitors can block multiple signaling pathways. JAK family kinases JAK1, JAK2, JAK3, and tyrosine kinase 2 (TYK 2) target a variety of cytokine pathways through cytokine receptors. JAK1, JAK2 and TYK2 are widely expressed in all kinds of cells, but the expression of JAK3 is limited within hematopoietic cells. They interact with the common gamma chain subunit of six cytokine receptors (IL-2, IL-4, IL-7, IL-9, IL-15, and IL-21) that have a crucial role in lymphopoiesis and homeostasis (112).

Tofacitinib is an oral small-molecule JAK inhibitor that can inhibit all JAKs, preferentially JAK1 and JAK3 and the efficacy of tofacitinib for the treatment of moderate to severe active UC has been approved (113). Sandborn et al. completed three-phase, randomized, and double-blind placebo-controlled trials of tofacitinib therapy in adults with UC and found that in the OCTAVE Induction 1 trial, the 8-week remission rate of tofacitinib induction therapy was 18.5%, higher than that of the control group (8.2%, *P* = 0.007) (105). In the OCTAVE Induction 2 trial, the 8-week remission rate was 16.6% in the tofacitinib treatment group vs. 6.6% in the control group (*P* < 0.001). In the OCTAVE Sustain trial, for UC patients with clinical responses to tofacitinib induction therapy, the 52-week remission rates of the 5 mg tofacitinib group, 10 mg tofacitinib group, and the control group were 34.3, 40.6, and 11.1%, respectively (*P* < 0.001, compared with the control group) (105). According to the study published recently, Tofacitinib has fast onset of action and seems to be effective even in cases of acute severe UC or refractory to anti-TNF-α (114, 115). The long-term safety of tofacitinib remains unclear, and the main side effects are herpes zoster virus infection and thrombosis (105). Therefore, clinical trials of other subtypes of selective JAK inhibitors are still ongoing to improve the benefit-to-risk ratio of JAK inhibitors.

Filgotinib is an oral selective JAK1 inhibitor. A phase 2, double-blind clinical trial of CD patients reported that 47% of the patients in the filgotinib group while 23% in the placebo group (*P* = 0.0077) had clinical remission at 10 weeks (106).

Upadacitinib is another selective JAK1 inhibitor. Sandborn et al. accomplished a phase 2b, multicenter and double-blind clinical trial of patients with moderate-to-severe refractory UC. No patients receiving placebo achieved clinical remission at week 8 and the rates of clinical remission in patients receiving 7.5, 15, 30, or 45 mg upadacitinib were 8.5, 14.3, 13.5, and 19.6%, respectively (*P* = 0.052, *P* = 0.013, *P* = 0.011, and *P* = 0.002, compared with placebo). Additionally, 14.9, 30.6, 26.9, and 35.7% of patients receiving 7.5, 15, 30, or 45 mg upadacitinib achieved endoscopic improvement (endoscopic subscore ≤ 1) at week 8, while only 2.2% of patients receiving placebo achieved endoscopic improvement (*P* = 0.033, *P* < 0.001, *P* < 0.001, and *P* < 0.001, compared with placebo, respectively) (107). In the 45 mg upadacitinib group, one patient developed herpes zoster, and another patient had pulmonary embolism and deep vein thrombosis. In addition, increased serum lipid levels and creatine phosphokinase were reported after upadacitinib treatment.

Deucravacitinib is a kind of highly selective TYK2 inhibitor and exerts less or no activity toward JAK3 (116). Deucravacitinib can significantly decrease the levels of IL-12 and IL-23 which may be helpful for IBD treatment (117). However, the clinical trials for deucravacitinib in IBD treatment are still ongoing and the efficacy and safety of deucravacitinib should still be better evaluated.

#### Sphingosine-1-Phosphate Receptor Modulators and Agonists

S1P is a lipid mediator which is derived from membrane sheath lipid metabolism. S1P is produced intracellularly and can be translocated to extracellular regions, where it plays a regulatory role in the immune system by activating specific receptors. Ozanimod (RPC1063) and Etrasimod (APD334), as S1P receptor (S1PR) modulator and agonist, are already being studied for the treatment of UC.

Ozanimod is an oral and selective S1PR modulator that acts on S1PR-1 and S1PR-5. It induces peripheral blood lymphocytes to isolate in the lymph nodes, thereby reducing the number of activated lymphocytes circulating to the inflammatory sites. Sandborn et al. first studied the effect of ozanimod on UC (108). This double-blind, placebo-controlled phase 2 trial showed that patients receiving ozanimod (1 mg/day) had a higher rate of clinical remission. In a recently published phase 3 multicenter randomized, placebo-controlled study, a total of 1,012 patients were included in the induction period and 457 patients in the maintenance period (109). Results showed a significantly higher clinical remission rate among patients receiving ozanimod than those receiving placebo during induction (18.4 vs. 6.0%, *P* < 0.001) and maintenance (37.0 vs. 18.5% among patients with a response at week 10, *P* < 0.001). The incidence of clinical response was also tremendously higher with ozanimod than with placebo during both the induction (47.8 vs. 25.9%, *P* < 0.001) and maintenance (60.0 vs. 41.0%, *P* < 0.001). Despite the risk of raised liver aminotransferase levels, ozanimod was more effective than placebo in inducing and maintaining the remission of moderately to severely active UC. Feagan et al. conducted a phase 2 prospective study to evaluate the effects of ozanimod in moderate to severe CD. After induction therapy for 12 weeks, 23.2, 39.1, and 56.5% of the patients experienced endoscopic response, clinical remission, and clinical response, respectively (110). Despite the lack of a contemporaneous control group, similar endoscopic and histopathology improvements to those in controlled trials of effective agents verified the therapeutic benefits of ozanimod. Additionally, the observer-blinded design of this study makes the results more persuasive.

Etrasimod, an oral S1PRS agonist, is selective for S1PR-1, S1PR-4, and S1PR-5. A phase 2 randomized, double-blind, placebo-controlled study was performed to assess the therapeutic effects of etrasimod for patients with moderately to severely active UC, which reported that patients receiving etrasimod at a daily dose of 2 mg achieved better clinical (*P* = 0.009) and endoscopic improvements (*P* = 0.003) than patients receiving placebo (111). There is no study reporting the treatment effects of etrasimod in CD. More studies are needed to further evaluate the efficacy and safety of ozanimod and etrasimod in IBD.

## Surgical Treatment

With the development of biologics, significant progress has been made in the drug treatment of IBD, but surgery is still an important means for IBD treatment. Despite the increased number of hospitalized patients in recent years, the rate of surgery for CD has decreased from 10 to 8.8% (*P* < 0.001), and that for UC has decreased from 7.7 to 7.5% (*P* < 0.001) (118). A study in the New York State Database demonstrated that in the era of biologics, the mortality rate of CD patients after non-selective surgery declined, but that of UC patients increased to 15% (119). There is still room for improvement in surgical and perioperative management.

The absolute indications of operation in UC patients principally include complicated massive bleeding, intestinal perforation, carcinogenesis and highly suspected carcinogenesis. Relative indications include: (1) Patients with severe UC fail to respond to active medical treatment, and patients with toxic megacolon have no response to medical treatment, earlier surgical intervention is suggested. (2) The medical treatment effect is poor and/or adverse drug reactions have seriously affected the quality of life (120).

For localized ileocaecal CD patients who failed to respond or relapse after initial medical therapy or preferred surgery to continued drug therapy, laparoscopic resection is recommended. Due to the poor long-term outcomes, surgical options for perianal Crohn's fistula can only be offered to a selected group of patients after consultation, especially patients with complex diseases and ongoing disease activity (120). One study showed that the symptomatic recurrence rate of CD patients was 20% at 1 year and 34% at 3 years after ileocolectomy, while the endoscopic recurrence rate reached 73 and 85%, respectively (121). Regular post-operative endoscopic examination may help monitor recurrence and develop prevention and treatment plans.

## Novel Therapies

Emerging therapeutic approaches, such as apheresis therapy, improved intestinal microecology, cell therapy, and exosome therapy, were reviewed in this section. More research on these therapies may provide new treatment options for IBD, bringing both opportunities and challenges.

### Apheresis Therapy

Apheresis therapy is a novel treatment for IBD developed in Japan, whose main mechanism is to reduce the local inflammatory response by isolating and absorbing one or more specific leukocytes (such as granulocytes, monocytes and activated lymphocytes) in the peripheral blood (122).

Adsorptive granulocyte/monocyte apheresis (GMA) has been shown to be effective in inducing remission in patients with UC and CD. A meta-analysis showed that GMA was more effective in inducing clinical remission in patients with active UC than CSs (OR = 2.23, 95% CI: 1.38–3.60) and that the incidence of adverse events associated with GMA was significantly lower than that with CSs (123). The efficacy (overall efficacy rate of about 70%) and safety of GMA were initially confirmed by a multicenter clinical trial in China that involved 34 patients with active UC (124). Motoya et al. found that the clinical remission rate of UC treated with GMA was 46.4% with no increase of adverse events in older patients with IBD (125). Recently, a retrospective analysis showed that nearly 80% of patients with UC achieved clinical remission after GMA treatment (126). Fukuchi et al. investigated the efficacy of GMA combined with TPs in the treatment of patients with early-diagnosed CD (127). The clinical remission rate and mucosal healing rate during the 52-week long treatment were 81.8 and 50%, respectively, without any serious adverse reactions.

GMA is quite popular among patients with IBD. About half of patients expressed their satisfaction with the effect of GMA after treatment, and 80% showed agreement to be treated with this technique again in the future, regardless of the response to the treatment (128). However, evidence for the effectiveness of GMA maintenance therapy is scarce and further studies are needed.

### The Improvement of Intestinal Microecology

Changes in the composition and function of the intestinal microbiota were found in patients with IBD (129). Although the specific mechanism of IBD remains unclear, the occurrence of IBD is found closely related to the imbalance of intestinal microecology. The imbalance between beneficial bacteria and harmful pathogenic bacteria in patients can trigger abnormal immune response in genetically susceptible people (129). Meanwhile, the inflammatory cells and factors can cause the intestinal mucosa injury. In IBD patients, the biodiversity of intestinal microbiota was decreased, with the most pronounced changes in the number of normal anaerobic bacteria such as Bacteroides, Eubacteria and Lactobacilli. Mucosal inflammation in IBD has been proved associated with the loss of these normal anaerobic bacteria (130).

On the theoretical basis of intestinal microbiota disorder, researchers have found potentially effective treatment methods for IBD by improving intestinal microecology with progressive achievements in recent years, including antibiotics, probiotics, prebiotics, postbiotics, synbiotics, and fecal microbiota transplantation (FMT).

#### Antibiotics

Researchers have made more efforts to explore the role of antibiotics in the treatment of IBD. Antibiotics are expected to be a future treatment choice for IBD, given their potential influence on the intestinal microbiota composition. A systematic review and meta-analysis published in 2011 demonstrated the positive effects of antibiotics for both UC and CD (131). Uchino et al. conducted a randomized controlled trial and proved that combined oral and intravenous antibiotics in CD patients could decrease the incidence of incisional infection (7.4 vs.16.6%, *P* = 0.01) compared with intravenous antimicrobial prophylaxis alone (132). They concluded the absence of oral antibiotics as an independent risk factor for surgical site infections and revealed the importance of preoperative oral antibiotics for CD patients. In the 3rd European evidence-based consensus, the use of antibiotics in CD patients is appropriate for septic complications, symptoms attributable to bacterial overgrowth or perineal disease (133). However, a Cochrane database systematic review in 2019 reported that the efficacy of antibiotics in inducing remission of CD appeared be modest and might not be clinically meaningful (134). The effect on the maintenance of remission and the risk of severe adverse events in CD was unclear. Moreover, studies on the effect of antibiotics in UC are insufficient to recommend antibiotics for induction or maintenance of remission (135).

It is important to note that using antibiotics may be an independent risk factor for IBD. Higher cumulative exposure to systemic antibiotic therapy, particularly treatments with a greater spectrum of microbial coverage, may be associated with a higher risk of new-onset IBD (136). Compared with patients taking no antibiotics, the patients taking antibiotics showed a OR value of 1.88 (95% CI: 1.79–1.98) for the occurrence of IBD, 1.74 (95% CI: 1.64–1.85) for UC, and 2.27 (95% CI: 2.06–2.49) for CD, respectively (136). Additionally, Shaw et al. found that changes in intestinal symbiotic bacteria caused by antibiotics use in infants and children were associated with the development of IBD (137). Of 36 children with IBD, 58% had received antibiotics within the first year of life while only 39% of the 360 normal children received that. Patients who received antibiotics within the first year of life were 2.9 times more likely to develop IBD than those who did (95% CI: 1.2–7.0). Moreover, Balram *et al*. found that antibiotics use within 30 days of diagnosis appeared to increase the risk of clostridium difficile infection (CDI) among IBD patients (OR = 1.85, 95% CI: 1.36–2.52) (138). Therefore, further emphasis should be attached to the management of antibiotics use.

#### Probiotics, Prebiotics, Synbiotics, and Postbiotics

Probiotics are live microorganisms that are intended to have health benefits when consumed or applied to the body. They can reduce epithelial cell apoptosis and attenuate intestinal mucosal inflammation (139). Probiotics are usually bacteria that produce lactic acid, which can be obtained by ingesting fermented foods, such as yogurt, fermented dairy products, and fermented byproducts of cured meats (140). Prebiotics can be health-promoting substrates selectively utilized by host microbes and supplemented by the intake of legumes, fruits, and vegetables. Generally, beneficial prebiotics includes polyols (sugar alcohols), oligosaccharides, and soluble fiber (141). Synbiotics are the synergistic combination of probiotics and prebiotics found in foods, drugs, and supplements (142–144). The main microbial-derived metabolites are postbiotics, including bile acids, short-chain fatty acids, and tryptophan metabolites (145).

Probiotics, prebiotics, and synbiotics have been proved beneficial in IBD, especially the combination ones in UC (140, 146). Subgroup analyses showed that synbiotics might be more effective than probiotics or prebiotics in inducing or maintaining IBD remission (140). Additionally, probiotics, prebiotics, or synbiotics in combination with conventional drugs were superior to conventional drugs alone (147). A randomized controlled trial demonstrated that regular consumption of kefir (a fermented probiotic dairy product) containing lactobacilli can modulate intestinal biota so as to improve quality of life in patients with IBD (148).

Multiple studies have shown the potential effects of probiotics in UC but not in CD (146, 149–151). Some studies found a beneficial effect of probiotics on active UC (149, 150), but the effect showed little or no difference in clinical remission compared to 5-ASA (149). Some studies suggested that probiotics were as effective as 5-ASA in preventing the recurrence of inactive UC but showed no benefit in inducing remission of active UC over placebo (151). Bjarnason et al. conducted a single-center, randomized, double-blind, placebo-controlled trial, and found a multi-strain probiotic (Symprove™, Symprove Ltd, Farnham, United Kingdom) might reduce the intestinal inflammation in patients with UC (152). Many studies found no significant effects of probiotics for the treatment of CD (153, 154).

Probiotics appeared to be safe and well-tolerated. In a systematic review and meta-analysis, there was no significant evidence to prove the risk for the overall side effects (RR = 1.35, 95% CI: 0.93–1.94) and for gastrointestinal symptoms (RR = 1.78, 95% CI: 0.99–3.20) was higher in IBD patients taking probiotics than in those exposed to placebo (155). Probiotic supplements that were based on Lactobacillus and Bifidobacterium or more than one strain were more likely to be effective for IBD remission. It suggested the dose of 10^10^-10^12^ CFU/day as a reference range for using probiotics to relieve IBD (140).

Postbiotics could act as immunomodulators and motivate anti-inflammatory response (156), suggesting that postbiotics may be a treatment for IBD. A study assessing the potential role of postbiotics in an *ex-vivo* organ culture model showed that potent postbiotics could protect healthy tissue against inflammatory attacks and concluded that postbiotics could be an effective and safe choice for acute IBD (157). More in-depth studies are needed to elucidate their role in the treatment of IBD.

#### FMT

FMT is a new therapy that transplants the functional micromicrobiota from the feces of healthy donors into the gastrointestinal tract of patients suffering from intestinal microbiome disorders to reconstruct the intestinal microecology and cure disease. FMT has been shown to be effective in the treatment of recurrent and refractory CDI with a high success rate of 90% (158). In recent years, the potential of FMT in the treatment of IBD has been further unleashed. The Australian consensus on the clinical use of FMT acknowledged for the first time the efficacy of FMT in inducing remission in patients with mild to moderate UC (159).

The main advantage of FMT lies in the complete ecosystem it provides from healthy individuals, including the full spectrum of microbial organisms, which may address intestinal microdysbiosis and dysfunction in patients with IBD (160). FMT has gained development and application for its efficacy and safety in the treatment of IBD. FMT was superior to placebo in achieving clinical remission at week 7 in a randomized study on patients with active UC (161). A meta-analysis of the efficacy of FMT in IBD demonstrated that the clinical remission rate in UC and CD patients receiving FMT was 33% (95% CI: 23–43%) and 52% (95% CI: 31–72%), respectively (162). Meanwhile, the association between FMT and clinical remission in patients with UC was discovered in a meta-analysis of 4 randomized controlled trials (OR = 2.89, 95% CI: 1.36–6.13).

Multiple studies confirmed that, the intestinal microbial diversity of the recipient increased after FMT (162–164) and showed a similarity to the microbiota of donor (162), thus giving great importance of the selection of donor because the intestinal microorganism status of the donor can affect the efficacy of FMT. It was found that the higher the microbial richness of the donor, the higher the success rate of transplantation treatment (163). A double-blind, randomized controlled trial reported that 27% of the patients allocated FMT and 8% of those assigned placebo achieved steroid-free clinical remission with endoscopic remission or response (RR = 3.6, 95% CI 1.1–11.9; *p* = 0.021) (165). A multicenter, randomized, double-blind clinical trial was conducted to evaluate the efficacy of FMT protocols in adults with mildly to moderately active UC using an anaerobically prepared stool. The results proved that patients receiving anaerobically prepared pooled donor FMT had a higher steroid-free remission rate (32%) than those (9%) receiving autologous FMT processed under aerobic conditions at week 8 (*P* = 0.03) (166).

The optimal route and regimen of FMT administration requires further study. A meta-analysis suggested that FMT administration via the lower gastrointestinal tract was more effective than the upper gastrointestinal tract in patients with UC (162). However, no unified standard has been made. Compared with traditional drug therapy, the time-consuming and labor-consuming colonoscopic FMT with the unknown safety of long-term frequent operation cannot be used as a routine choice for IBD treatment. The emergence of encapsulation methods, such as liquefaction, freezing, and freeze-drying, provides new ideas for the application of FMT in the maintenance treatment of IBD. The clinical efficacy of oral capsule FMT in the treatment of refractory CDI has been validated (167–169).

Combing FMT and antibiotics may improve the efficiency of IBD treatment. A meta-analysis demonstrated that patients with UC who received antibiotics before FMT had a higher rate of clinical remission than patients who did not before FMT (54 vs. 25%, *P* = 0.03) (170). However, it remains unclear how to select the appropriate antibiotics or combinations of antibiotics for different patients to achieve the optimal intestinal microecology after antibiotic treatment and FMT treatment. Bacteriological and metabolic analyses of fecal samples before and after FMT revealed that compared to patients who did not achieve remission, patients achieving remission after FMT had higher concentrations of Eubacterium hallii and Roseburia inulivorans and increased levels of short-chain fatty acid biosynthesis and secondary bile acids.

There are challenges before the application of FMT in the treatment of IBD: (1) The long-term efficacy is unknown: most clinical studies' period is short with the longest ones lasting 1 year in the treatment of UC (161, 166). (2) The safety is unknown: the reported side effects of FMT treatment in IBD patients include common gastrointestinal reactions (e.g., bloating, diarrhea, and abdominal pain) (162), complications related to administration route (e.g., aspiration pneumonia and intestinal perforation) (163, 171), as well as IBD related ones (e.g., toxic hypercolon and sepsis) (162). However, few long-term statistics on the safety of FMT in patients with IBD have been collected. (3) The feasibility of universal implementation and management is unknown: the lack of unified standards for the use of FMT in IBD around the world limits its further application (172).

In summary, FMT is expected to be a new option for IBD treatment. Future research should focus on the donor-receptor matching based on microbial characterization (165), selection of administration routes, and determination of optimal intensity of treatment. Meanwhile, additional preclinical studies and clinical trials are necessary to provide data on the long-term efficacy and safety of FMT.

### Stem Cell Transplantation

Stem cells can differentiate into more than one type of cells in the body and keep dividing and proliferating (173). Stem cell transplantation can promote the regeneration of injured tissue and help restore specific tissue functions, thus restoring the integrity of the intestinal mucosal barrier in patients with IBD. In recent years, advances in stem cell biology have opened new grounds for the application of stem cells of different types in the treatment of IBD.

The cells involved in the pathophysiological process of IBD include inflammatory cells of the lamina propria, intestinal mesenchymal cells and intestinal epithelial cells (IECs). Therefore, haematopoietic stem cells (HSCs), mesenchymal stem cells (MSCs), and intestinal stem cells (ISCs) are candidates for IBD cell therapy.

#### HSCs Transplantation

HSCs have the ability to migrate to injured tissues and facilitate tissue renovation, renewal, and regeneration (174, 175). We usually choose autologous HSCs for HSCs transplantation (HSCT) because of gastrointestinal disorders and even the occurrence of IBD (176, 177) after allogeneic HSCT. Autologous HSCT rebuilds the host's immune system by generating new self-tolerant lymphocytes after chemotherapy-induced elimination of self- or auto-reactive lymphocytes (178). The tissue sources of HSCs for therapeutic use are mainly derived from bone marrow, umbilical cord and peripheral blood, with the most specific marker of the cell surface glycoprotein CD34.

HSCT is the most widely used cell regeneration therapy for its feasibility in clinical practice. Early reports showed that 2 patients with severe CD who had no response to anti-TNF-α therapy achieved clinical remission and maintained for more than 1 year after large dose of immunosuppression and autologous HSCT (179). A European retrospective study of 82 patients with severe refractory CD showed 68% of patients had complete remission or significant improvement in symptoms after treatment with autologous HSCT, with a median follow-up of 41 months (6–174 months) (180). Lindsay et al. found that within 1 year after autologous HSCT, 38% of patients with CD achieved a 3-month steroid-free remission, and about half of these patients achieved mucosal healing (181). However, the safety and long-term efficacy of HSCT in the treatment of IBD call for further discussion. A retrospective study reported the unpromising long-term remission of autologous HSCT because most patients required salvage or maintenance treatment within 1 year after autologous HSCT (182). A cohort study showed that although the majority of CD patients relapse within 5 years after autologous HSCT, 80% of relapsed patients returned to clinical remission after re-treatment with HSCT (183). In addition, infectious adverse events [viral infection (180), sepsis, and pneumonia] were common within 100 days after autologous HSCT (184). Some researchers believe that autologous HSCT with low intensity is safe and effective for refractory CD (185). In a study with 14 refractory CD patients, autologous HSCT with low-dose cyclophosphamide was used (186). Compared with previous studies using high-dose cyclophosphamide, the duration of anemia and neutropenia was shorter. The lower intensity cyclophosphamide still achieved an effective treatment for refractory CD, with 13 patients achieving disease remission at 30 days.

In conclusion, although many studies have confirmed the efficacy of HSCT in the treatment of some patients with refractory CD, caution should be exercised due to the high risk of adverse events after HSCT. In addition, the number of HSCs is limited, accounting for only 1/100,000 (187). Meanwhile, patients' unique conditions must be fully considered before treated with autologous HSCT.

#### MSCs Transplantation

MSCs are widely distributed in various tissues (bone marrow, peripheral blood, fat, skeletal muscle, etc.). They have strong ability to proliferate and can differentiate into various mesodermal cell types (adipocytes, osteoblasts, or chondrocytes) under specific induction conditions *in vitro* (188). Due to the low immunogenicity of MSCs, allogeneic MSCs transplantation can be used safely without immunosuppression (178). The superiority of MSCs also lies in their tissue-specific differentiation, strong immunomodulation, and plentiful trophic factor production (178).

The main tissue sources of MSCs used for treatment are bone marrow, umbilical cord and adipose tissue, and other sources like amniotic membrane and fetal membrane. Both adipose-derived stem cells (ADSCs) and bone marrow mesenchymal stem cells (BMSCs) showed the morphological and immunophenotypic characteristics of MSCs, with positive expression of MSCs markers and negative expression of hematopoietic markers (189). Compared to BMSCs, ADSCs can secrete higher levels of anti-inflammatory cytokines (IL-6 and TGF-β) involved in immune regulation. Therefore, ADSCs may have stronger immunomodulatory ability than BMSCs (190).

A phase 1 clinical study demonstrated the safety of autologous BMSCs transplantation in patients with refractory CD (191). A phase 2 clinical study showed that after intravenous administration of allogeneic BMSCs for 4 weeks, 80% of patients with refractory CD had a clinical response, and over half of the patients achieved clinical remission. Additionally, 47% of the patients had endoscopic improvement, with a low incidence of adverse events (192). Dietz et al. explored the efficacy of autologous MSCs on perianal fistula of patients with CD through a phase 1 trial and recorded a clinical cure rate of over 80% at 6 months (193). A phase 3 clinical trial demonstrated that the healing rate of complex anal fistulas was ~40% after 6 months of allogeneic ADSCs transplantation (194). A meta-analysis and systematic review reported that patients receiving MSCs transplantation had a higher rate of fistula healing than patients receiving placebo (61.75 vs. 40.46%, *P* < 0.05). MSCs transplantation, especially ADSCs, was well-tolerated with a lower incidence of adverse events than placebo (195).

MSCs regulate immune response by down-regulating the levels of pro-inflammatory cytokines (TNF-α, IFN-γ, 1L-12, IL-17, etc.) and up-regulating the level of anti-inflammatory cytokines (IL-10). MSCs directly target the Th-17 cells and increase their expression of FoxP3 mRNA, thus switching them into Tregs and inhibiting their production of inflammatory cytokines. MSCs can also inhibit the Th1-driven autoimmune response (196–198). The effect of donor MSCs on the recipient is mainly through paracrine release of various cytokines (199), which not only participate in the regulation of immune response but also promote tissue repair (178). Intraperitoneal BMSCs in mouse model of colitis localize to the peritoneum and produce sufficient immunoregulatory molecules there (200). Therefore, targeted transplantation is not necessary for MSCs transplantation to work.

MSCs transplantation with immunomodulatory effects can promote intestinal epithelial remodeling, which is expected to be an effective method for the treatment of IBD. Available clinical data have shown the potential therapeutic effect of MSCs transplantation on IBD and its complications, but MSCs have not been approved for clinical use. Therefore, more randomized controlled studies are needed to provide data support for the application of MSCs transplantation.

#### ISCs Transplantation

Intestinal epithelium consists of villi and crypt which is renewed every 2–6 days in healthy individuals due to the continuous proliferation of ISCs at the base of the intestinal crypt. ISCs have the ability to regenerate and differentiate into different types of IECs, such as goblet cells, endocrine cells, tuft cells, and absorptive cells. IBD damages the intestinal epithelium. Therefore, researchers hope to regenerate and repair the damaged intestinal epithelium through the transplantation of ISCs cultured *in vitro*, thus promoting the mucosal healing of patients with IBD. However, culturing ISCs *in vitro* remains a challenge in ISCs transplantation.

Sato et al. developed a technique for long-term culture of ISCs *in vitro* by culturing ISCs in a 3D structure named organoid that mimics the ISCs niche environment (201). The ecology of the ISCs *in vivo* was simulated by providing appropriate growth factors and the required extracellular matrix *in vitro* (202). The source of ISCs specimens for *in vitro* culture were the biopsy tissue of patients during endoscopic examination (203). A study reported that the position-specific function of adult ISCs was inherently programmed, and their differentiation fate was independent of position-specific extracellular signaling (204). These findings provided an important basis for the use of ISCs as donor cells for cell transplantation therapy. Another major breakthrough supporting the clinical application of ISCs is that organoids grown *in vitro* can be transplanted and integrated into the recipient intestinal epithelium. Yui et al. transplanted the donor organoid cells into the colon of the dextran sulfate sodium (DSS)-colitis mouse model using the intraluminal transplantation method. Those cells engrafted and covered the lesions in recipient mice and constituted a single-layered epithelium, which formed self-renewing crypts that were functionally and histologically normal (205).

However, several difficulties need to be overcome when treating IBD with ISCs. First of all, research on ISCs transplantation for IBD is still in its infancy with no clinical trials and limited repeated animal experiments. Secondly, many issues need to be addressed before establishing an accurate *in vitro* culture scheme of human ISCs. Thirdly, it is not clear at the moment whether autologous or allogeneic transplantation of ISCs is favorable to treat IBD patients. Moreover, methods to deliver the donor ISCs efficiently to the desired site, e.g., endoscopic technology, need to be established. Finally, the conditions under which patients can receive transplantation and the contraindications to the procedure need to be specified.

Development in stem cell biology has improved the feasibility of the culturing ISCs *in vitro*. However, there are many issues to address before establishing a safe and effective transplantation scheme for ISCs cultured *in vitro* to patients with IBD.

### Exosome Therapy

Exosomes are nanoscale microvesicles released from various types of cells and are widely distributed in biological fluids, which contain important regulatory factors that act on adjacent or distal cells through the systemic system and function in a variety of biological signaling pathways. It has been demonstrated that exosome-mediated immune responses play an important role in the pathogenesis of IBD (206).

The lipid bilayer of exosomes encases various functional components but no organelles. Their functions mainly depend on their internal functional components, like proteins, nucleic acids, and other substances. The structure of exosomes themselves is also important. Studies have shown that exosome structure can increase the stability of internal biological components (207, 208). Exosomes are involved in numerous physiological processes, such as immune regulation, tissue repair, and regeneration (209). Therefore, exosomes have great clinical potential in the treatment of IBD.

The main sources of exosomes are stem cells, immune cells, IECs, body fluids, food and parasites. Stem cell-derived exosomes contribute to stem cell self-renewal, injury repair, and immune regulation. Mao et al. found that exosomes released by MSCs in human umbilical cord blood could reduce inflammatory response in mouse model of IBD and contribute to the recovery of damaged tissues and organs (210). Liu et al. further confirmed the therapeutic effect of MSCs-derived exosomes on IBD and deemed that the therapeutic effects depended on macrophages (211). Exosomes derived from immune cells (such as macrophages, monocytes, and dendritic cells) can evade clearance by the immune system, thereby prolonging their cycle and duration of action. It was reported that exosomes produced by IL-10-treated dendritic cells inhibited colitis of mouse model (212). Another study suggested that TGF-β1 gene-modified exosomes alleviated colitis in mouse model (207). Exosomes produced by IECs are crucial to IECs-induced immune tolerance (206). Though exosomes can be isolated from blood (213), amniotic fluid (214), urine (215), and breast milk (216), their relevance to IBD treatment drawn inadequate academic attentions in the past. However, exosomes derived from food have gained recent popularity. Xiao et al. isolated exosome-like nanoparticles from 11 vegetables and fruits (217) and Zhao et al. isolated exosomes from coconut water (218). A study proved that milk could be the carrier of chemical drugs or other biological components with a targeted effect similar to exosomes (219). Recently, it has been found that extracellular vesicles (EVs) secreted by whipworm can interact with host cells and participate in the regulation of inflammation and immunity (220). EVs produced by hookworm can inhibit the production of inflammatory cytokines (IL-6, IL-1β, IFNγ, and IL-17), thereby alleviating colitis in mouse model (221).

Epithelial restitution is essential for barrier function repairment at injured mucosal surfaces. Prolonged breaches in epithelial barrier function result in inflammation and further damage. Endogenous annexin A1 (ANXA1) in exosomes can heal damaged intestinal epithelium through transducing the formyl peptide receptor signaling pathway (222). Patients with active IBD have higher levels of serum EVs containing ANXA1 than healthy controls (222). Polymeric nanomaterials containing exogenous ANXA1 mimetic peptides can target the impaired intestine and accelerate the intestinal healing and the recovery of intestinal epithelial barrier function in mouse model of UC (222). Heat shock proteins (HSPs) are important elements in the body's defense against various damaging factors. It has been shown that HSPs in exosomes, such as HSP20, HSP27, HSP70 family, and HSP90, are involved in the pathogenesis of IBD (223, 224). Cellular prion protein (PrPc) is a glycosylphosphatidylinositol-anchored glycoprotein ubiquitous in the cellular junctions of many tissues. PrPc has also been found in platelet-released exosomes (225). A study found reduced PrPc levels at the epithelial cell-cell junction of the colon in patients with IBD (226). PrPc regulates IEC-cell junction and plays an important role in maintaining intestinal barrier function (226). Exosomes isolated from vegetables and fruits have anti-inflammatory properties, and their internal miRNAs (small non-coding RNAs) can regulate human mRNA (217).

Design of new drug formulations using exosomal structures may provide new insights for the treatment of IBD. Both animal and clinical studies are required to verify the efficacy of exosomes on IBD before clinical use.

### Others

There are also many emerging therapeutic methods for IBD treatment. ABX464, as a novel drug candidate for human immunodeficiency virus (HIV), has shown strong antiviral properties (227). Recent studies have discovered that ABX464 can upregulate the expression of miR-124, thus inhibiting the inflammatory response for IBD treatment (228, 229). A phase 2 study in moderate to severe UC patients treated with oral administration of ABX464 or placebo was conducted (230). After treating 8 weeks, the difference of the endoscopic improvement was significant between ABX 464 (50 mg daily) group and placebo group (*P* < 0.05). In addition, the overall safety of ABX464 was satisfactory with no obvious side effects. Mongerson, as an oral anti-sense small oligonucleotide, can decrease the translation of SMAD7, which will result in the anti-inflammatory response of TGF-b on mucosa (231). A clinical trial indicated that the administration of Mongerson (40 and 160 mg daily) for 2 weeks was better than placebo at inducing the remission of CD (232). However, the longer efficacy and safety of Mongerson for IBD treatment still need further investigation. IL-10 is a kind of proinflammatory cytokine that can decrease a number of proinflammatory signals associated with IBD (233). However, the application of IL-10 has been limited by some side effects (*e.g*., anemia and thrombocytopenia) and gut-restricted distribution. AMT-101 is a chimera produced by genetically fusing non-toxic fragment of cholix to human IL-10 (233). AMT-101 can efficiently cross the epithelial barrier and selectively activate human IL-10 receptors in the intestinal lamina propria (233), which allows IL-10 to play a targeted anti-inflammatory role without causing systemic side effects. Further clinical studies are needed to evaluate the efficacy and safety of AMT-101 for IBD treatment.

## General Measures and Education

There are several general measures for prevention and treatment of the complications in patients with IBD. Many factors within the patient may influence the outcome of IBD treatment, including diet, mood, and other lifestyle factors. In addition, education is necessary for patients and could help patients manage these factors scientifically.

### General Measures

Patients with IBD are vulnerable to water and electrolyte balance disorders and malnutrition and there are severe cases such as chronic anemia, and high homocysteinemia that threaten the patients' life. Therefore, appropriate symptomatic treatment measures are necessary. Disordered water and electrolyte balance and acid-base balance should be corrected. Anemic patients should be transfused. Patients with hypoproteinemia should be injected with human albumin. Body mass index (BMI), iron, calcium, and vitamins (especially vitamin D and B12) should be monitored and adjusted accordingly. Nutritional support treatment should be given to patients with severe illnesses. Enteral nutrition is the first choice, and parenteral nutrition can be supplemented if enteral nutrition is insufficient (234). Patients with abdominal pain and diarrhea should take anticholinergic drugs or antidiarrheal drugs when necessary. Patients with severe poisoning symptoms should be given broad-spectrum antibiotics by the intravenous route.

IBD has been proved an independent risk factor for recurrence VTE (RR = 2.5; 95% CI: 1.4–4.2; *P* = 0.001) (235). Because of the high morbidity and mortality of VTE, thromboprophylaxis is essential, which is mainly achieved by correcting risk factors and using drugs. Correcting risk factors refers to controlling disease activity and avoiding long-term bed rest. Low molecular weight heparin is recommended for drug prophylaxis. However, thromboprophylaxis in IBD patients has not been well-implemented due to the lack of awareness or safety concerns.

### Education

#### Diet

Diet alters the composition of the gut microbiome and the production of absorbable metabolites (236), which are important messengers in the interactions among diet, the gut microbiome, and the host (237). As a result, diet may affect the disease activity, symptoms and prognosis of IBD.

Certain components of the diet have anti-inflammatory or pro-inflammatory properties and will affect the course of IBD. Dietary inflammatory index (DII) is an index to quantify the potential inflammatory effect in the diet, which reflects a large literature and population base, and is associated with international standard (238, 239). A recent analysis found that dietary patterns with a high DII could increase the risk of CD (240). It has been shown that DII is positively correlated with disease activity in CD patients and there is no correlation between DII and disease activity of UC (241). However, a study of Iranian patients with IBD indicated that there was no association between DII and disease activity, which may be due to the small sample size (*n* = 143) or influence of other variables (242).

Exploring the influence of dietary interventions on IBD disease activity helps provide dietary guidelines for patients. An review reported that more than half of IBD patients were deficient in micronutrients, such as iron, vitamin B12, vitamin D, vitamin K, and folic acid (243). It has been proved that the supplementation of Vitamin D, which can modulate the immune response and reduce inflammation, may improve outcomes of the treatment of patients with IBD as the active component of vitamin D [1,25-(OH)D3] can interact with T cells and regulate immune response mediated by T cells (244). Vitamin D can also inhibit the inflammatory activity of dendritic cells, induce antimicrobial activity and regulate the production of cytokines to enhance the anti-inflammatory effect (244). In addition, vitamin D supplementation can help increase bone density and reduce the risk of fracture in IBD patients. However, vitamin D is a fat-soluble vitamin and should not be overused. Further research is needed to determine the optimal serum vitamin D levels for optimal therapeutic effects (244).

One study found that CD patients on a high-fiber diet were 40% less likely to have disease recurrence within 6 months than those on a low-fiber diet (245). However, a Cochrane review in 2019 analyzed 18 studies and indicated that the effects of dietary interventions, including high fiber, low refined carbohydrates, low microparticle, low calcium, symptoms-guided diet, highly restricted organic and low red processed meat diets, on CD and UC were uncertain (246). Albenberg et al. implemented a randomized controlled trial and confirmed that low red processed meat diet couldn't reduce the relapse rate in patients with quiescent CD (247). One of the most frequently used elimination diets in CD patients is the diet of low-FODMAP (fermentable oligosaccharides, disaccharides, monosaccharides, and polyols). Recently, a single-blind, randomized and controlled trial performed by Cox et al. initially demonstrated that the low-FODMAP diet made significant differences among patients with CD in remission, in symptom relief and improvement of life quality, but not in irritable bowel syndrome severity scores (248). Studies revealed that in stool samples collected at the end of the study period, patients on the low-FODMAP diet had a significantly lower abundance of Bifidobacterium adolescentis, Bifidobacterium longum, and Faecalibacterium prausnitzii than patients on the control diet. Bifidobacteria and Faecalibacterium prausnitzii, which have immune-regulatory effects, can increase peripheral blood mononuclear cell IL-10 production *in vitro* (249, 250). Faecalibacterium prausnitzii showed anti-inflammatory effects and was associated with lower post-operative ileal CD recurrence (250). Despite this, there were no detrimental effects of a low-FODMAP diet on patients with quiescent IBD (248).

Mediterranean-style diet (DINE-CD) is another kind of dietary intervention for IBD treatment. DINE-CD requires a diet high in omega-3′s and low in omega-6′s, which may reduce intestinal inflammation (251). A study found that too much intake of meat, omega-6′s fatty acids, and total fats resulted in high risks of IBD (252). In this study, the benefits of fruits and vegetables and the disadvantages of high meat and fats intake indicate the promise of DINE-CD for IBD treatment. However, some researchers found that adherence to DINE-CD was very low for IBD patients (253). Significantly, these patients would like to extend their nutritional knowledge for a better remission effect.

There is a deficiency of well-designed randomized controlled trials in this area and further research is needed to investigate the effects of various dietary interventions on IBD patients.

#### Mood and Psychology

IBD patients may have abdominal pain, diarrhea, mucinous blood, and other symptoms. The recurring symptoms and long-term medication will bring a heavy economic burden to the family, so IBD patients are prone to anxiety, depression and other adverse emotions. Patients and their families should be positively guided to have a full understanding of the disease. They are expected to be psychologically prepared and learn to correctly deal with the symptoms. Patients in the active phase of disease should have adequate rest, vent their negative emotions properly, and avoid excessive psychological pressure. They are encouraged to communicate with others and cooperate with treatment to reduce the recurrence of the disease.

#### Others

A systematic review has demonstrated that for patients with CD, smoking cessation can reduce recurrence risk by 65% compared with continued smokers. Smoking can reduce the drug efficacy and increase surgical and post-operative recurrence rate (254). In addition, patients should take medication as prescribed and participate in regular follow-ups.

## Discussion

At present, the treatment of IBD is primarily pharmacological, consisting mainly of aminosalicylates, CSs, immunomodulators, and biologics. However, a considerable number of patients fail to achieve clinical remission after treatment, or lose response over time. Additionally, more clinical data on the long-term safety of drugs are required. With the deepening of research, new therapies for IBD treatment are coming into view, mainly including apheresis therapy, improvement of intestinal microecology, stem cell transplantation, and exosome therapy. These non-approved novel therapies are often applied in investigational protocols, but are limited by their unclear impact on IBD. We still confront with many unresolved challenges before applying these emerging treatment options into clinical management. More research including long-term data are required to minimize the risk and optimize the treatment outcomes.

With the application of biologics, the therapeutic objectives of IBD have changed, and a new concept named mucosal healing has become well-known. It should be noted that histological mucosal healing is different from endoscopic mucosal healing. In cases of endoscopic remission, histological inflammation may persist and be associated with adverse outcomes. Endoscopy maintains a key role in monitoring mucosal healing nowadays. Histological assessment has not been widely used because of the lack of a valid valuation system and complex heterogeneity of disease (255). Nevertheless, histological mucosal healing has the potential to become a higher therapeutic target, considering recent developments in histologic assessment tools in UC (256).

With growing appreciation for mucosal healing, a treat-to-target strategy has gained wide acceptance. In order to verify the progress realized in the therapeutic path, an objective evaluation of mucosal inflammatory response is crucial. In the past, clinical symptom scores (such as CD activity index) were used to evaluate the treatment efficacy. Nowadays, endoscopy, histology, radiology, immunobiochemical monitoring biomarkers, quality of life assessment, and other methods have been introduced to provide more valuable references for the assessment of disease activity. There is no doubt that a multidisciplinary team, in which strong coordination between doctors, other health professionals (technicians, radiologists, biologists) and the patients is needed. Studies have shown that compared with focusing on clinical symptoms alone, targeting mucosal healing or inflammation control appears to be more cost-effective (257).

In the progress of achieving personalized and precise therapy, there are both opportunities and challenges. Doctors should fully grasp the indications, contraindications, as well as evidence-based medicine of various drugs and treatments, so as to develop individualized treatment plans based on the comprehensive assessment of the patient. The treatment should be flexible and changed according to the patient's response to the treatment. Additionally, self-management and regular follow-up of patients should not be neglected. Timely communication and close cooperation between doctors and patients are equally essential to effective treatment strategies. All the above play a necessary role in the induction and maintenance of remission in patients with IBD.

## Author Contributions

ZC performed the literature search and wrote the paper. SW revised the paper. JL designed the review and revised the paper. All authors read and approved the final manuscript.

## Funding

This study was supported by grants from the Science and Technology Development Project of Jilin Province (#3D5197434429 and 2021LC019), the Youth Program of the National Natural Science Foundation of China (#3A4205367429), and the Education Project of Jilin University (#419070600046).

## Conflict of Interest

The authors declare that the research was conducted in the absence of any commercial or financial relationships that could be construed as a potential conflict of interest.

## Publisher's Note

All claims expressed in this article are solely those of the authors and do not necessarily represent those of their affiliated organizations, or those of the publisher, the editors and the reviewers. Any product that may be evaluated in this article, or claim that may be made by its manufacturer, is not guaranteed or endorsed by the publisher.
