# Supplementary material for: Treatment of Inflammatory Bowel Disease: A Comprehensive Review
Source: Front Med (Lausanne). 2021 Dec 20;8:765474. doi: 10.3389/fmed.2021.765474 (PMC8720971; doi:10.3389/fmed.2021.765474)
Supplement: Supplementary file 1 [file Data_Sheet_1.docx]

| **Supplementary Table 1. Immunomodulators** | | | | | | | |
| --- | --- | --- | --- | --- | --- | --- | --- |
| Type of study | Patients | Treatment | Median treatment duration | Median follow-up duration | Results/ Conclusion | Adverse events | Reference |
| A retrospective study | UC and CD patients | TPs | NA | NA | AZA and 6-MP were effective in approximately 40% of patients after 5 years of treatment. More than a quarter of the patients discontinued TPs within 3 months, mainly due to adverse events | Hepatotoxicity, gastrointestinal complaints, myelosuppression, pancreatitis, fever, general malaise, arthralgia | (44) |
| A prospective, long-term, follow-up study | UC and CD patients | AZA (2.5 mg ⁄ kg) | 38 months | 38 months | AZA was effective for long-term treatment of IBD, and reduced the hospitalization and surgery rates. The effect was similar for UC and CD | Gastrointestinal intolerance, myelotoxicity | (45) |
| An open-label retrospective study | UC patients | TP monotherapy | NA | 36 months (1-210 months) | About half of patients achieved steroid-free remission. TP maintenance treatment was effective and safe for UC patients | Gastrointestinal intolerance, leukopenia, elevation of serum transaminases and pancreatic enzymes, infections | (46) |
| A systematic review and meta-analysis | UC patients | AZA/ MP | NA | NA | TPs were effective for the induction and maintenance of remission | NA | (47) |
| A retrospective observational cohort analysis | UC patients who achieved remission after induction with drugs other than biologics | Maintenance treatment with TPs and 5-ASA | NA | NA | The 7-year cumulative remission-maintenance and colectomy-free survival rates were 43.9% and 88.0%, respectively. TPs were of great benefit and well-tolerated in the long-term treatment | Infectious mononucleosis, nausea, liver dysfunction, pancreatitis, leukopenia | (48) |
| A prospective, observational, longitudinal follow-up study | Adult steroid-dependent patients with CD | AZA | 83 months | 10 years | 70% of patients treated with AZA maintained steroid-free remission for 5 years, which provided an option for the long-term management of steroid-dependent CD | NA | (49) |
| An uncontrolled study | Patients with refractory CD | MTX (25 mg/week for 3 months, then tapered to a  minimum of 7.5 mg/week) | 5.5 months (2 weeks -24 months). | NA | MTX appeared to be beneficial in short-term treatment, but the long-term benefit seemed limited. The side effects of MTX remained moderate | Gastrointestinal intolerance, abnormal liver function tests, headache, dizziness, fatigue, infections, gynaecomastia, skin rash | (53) |
| A double-blind, placebo-controlled, multicenter study | Patients with chronically active CD who had entered remission after 16 to 24 weeks of treatment with 25 mg of MTX given intramuscularly once weekly | Intramuscular MTX (15 mg once weekly) | 40 weeks | 40 weeks | MTX was effective for the maintenance of remission in the treatment of CD | Nausea and vomiting, symptoms of a cold, abdominal pain, headache, joint pain, arthralgia, fatigue | (54) |
| A Cochrane database systematic review | Patients with active UC | MTX | NA | NA | No significant difference in clinical remission rates between MTX and placebo patients were found | Nausea, dyspepsia, mild alopecia, mild increase in aspartate aminotransferase levels, peritoneal abscess, hypoalbuminemia, severe rash | (55) |
| A randomized, double-blind, controlled trial | Patients with severe UC refractory to CSs | Intravenous CsA (4mg/kg body weight per day) | 14 days | NA | Intravenous CsA was effective in the induction therapy for patients with severe corticosteroid-resistant UC | Paresthesias, hypertension, nausea and vomiting, a grand mal seizure | (58) |
| A single center, randomized, double-blind, controlled trial | Patients with severe UC | Intravenous CsA (4 mg/kg or 2 mg/kg body weight per day) | 8 days | NA | Low-dose intravenous CsA may be less toxic while has a similar therapeutic efficacy with high-dose CsA | Neurologic adverse effects (tremor or paresthesia), hypertension, increase serum creatinine (>10%), fever, diabetes mellitus | (59) |
| A prospective, multicenter, double-blind, and placebo-controlled trial | Patients with chronic active CD | CsA (5 mg/kg/day) | 12 months | 12 months | CsA plus low-dose steroids did not offer an advantage for the long-term outcomes compared with low-dose steroids solely | Renal dysfunction, depression, septicemia, hypertrichosis, paresthesia, headache, nausea, bronchopneumonia | (60) |
| A randomized double-blind, placebo-controlled, study | Patients with refractory active UC | Oral TAC (FK506) | 2+10 weeks | 12 weeks | The efficacy of oral TAC in inducing remission was dose-dependent. The optimal serum trough level appeared to be 10–15 ng/ml in terms of efficacy with two-week therapy | Tremor finger, hot flush, sleepiness, gastroenteritis, sepsis | (63) |
| A retrospective, observational, single-center study | Patients with UC who were resistant to or could not be treated with conventional therapy | TAC with trough whole-blood levels of 10-15 ng/mL to induce remission and 5-10 ng/mL to maintain remission. | 11 months (1–39 months) | 17 months (2–65 months) | TAC appeared to be effective and relatively safe in the long-term treatment of refractory UC | Tremor, renal function impairment, infections, hot flashes, hyperkalaemia, headache, epigastralgia, nausea | (64) |
| A clinical trial | Patients with active, moderate/severe steroid-refractory UC | Oral TAC (FK 506) (0.15 mg/kg/day) | 12 weeks | 21 months | Oral TAC represented effective for steroid-refractory UC, but its side effects were expected to be watched carefully | Thrombopenia, bicytopenia (anemia and leukopenia), fever, paresthesia, mild alopecia, verrucae, intermittent hypomagnesemia | (65) |
| A retrospective, observational single center study | Adult patients with steroid-dependent or steroid-refractory IBD | TAC (the serum trough levels was adjusted  to 4–8 ng/mL) | 25.2± 4.6 SD months (0.43–164 months) | 39±4.1 SD months (5–164 months) | Long-term TAC therapy appeared to be safe and effective in refractory IBD | A temporary rise of creatinine, tremor, paresthesias, hyperkalemia, hypertension, opportunistic infections | (66) |
| A systematic review and meta-analysis | Patients with active UC | TAC | NA | NA | TAC was associated with high clinical response and colectomy-free rates without increased risk of severe adverse events for active UC | NA | (67) |
| A systematic review | CD patients | TAC | NA | NA | TAC seemed beneficial for CD, although nonserious adverse effects were common | Tremor, paraesthesia, headache, nephrotoxity | (68) |
| A systematic review and meta-analysis | CD patients | TAC | NA | NA | TAC was effective and well-tolerant for CD patients | Temperature Cre rising, tremor, paresthesia, headache, nausea, tremor, insomnia, pruritus, diarrhea, leg cramps, irritability | (69) |
| A retrospective cohort study | Patients with severe UC | Intravenous CsA or oral TAC | 1 year | 1 year | Among patients who were previously exposed to biologics, the use of calcineurin inhibitors resulted in significantly higher 1-year colectomy rates when compared to patients who were biologic-naïve | NA | (70) |

*UC, ulcerative colitis; CD, Crohn’s disease; TP, thiopurine; NA, not applicable; AZA, azathioprine; MP, mercaptopurine; IBD, inflammatory bowel disease**; 5-ASA, 5-aminosalicylic acid; MTX, Methotrexate; CsA, Cyclosporine A; CSs, corticosteroids; TAC, Tacrolimus.*

| **Supplementary Table 2. Biologics** | | | | | | | |
| --- | --- | --- | --- | --- | --- | --- | --- |
| Type of study | Patients | Treatment | Median treatment duration | Median follow-up duration | Results/ Conclusion | Adverse events | Reference |
| A randomized, double-blind, placebo-controlled study | Patients with moderately to severely active UC | IFX (5 or 10 mg/kg at weeks 0, 2, and 6, then every 8 weeks through week 46) | 54 weeks | 54 weeks | The cumulative incidence of colectomy reduced by 7%. Fewer UC-related hospitalizations and surgeries/procedures occurred. | Infections, cancer, colonic dysplasia, optic neuritis, multifocal motor neuropathy | (72) |
| A randomized, multicenter, double-blind, placebo-controlled trial | CD adults who had draining abdominal or perianal fistulas of at least 3 months’ duration | Intravenous IFX (5 mg/kg or 10 mg/kg at weeks 0, 2, and 6) | 6 weeks | NA | Rates of response in patients given 5 mg/kg or 10 mg/kg IFX were 68% and 56%, respectively. after intravenous. 55% of the patients receiving 5 mg/kg IFX and 38% of those assigned to 10 mg/kg had closure of all fistulas. The median length of time during which the fistulas remained closed was 3 months | Headache, abscess, upper respiratory tract infection, fatigue | (73) |
| A phase 3, double-blind trial | Patients with moderate-to-severe UC who completed golimumab induction trials | Subcutaneous Golimumab (50 or 100 mg every 4 weeks through week 52) maintenance therapy | 52 weeks | 54 weeks | Golimumab helped to maintain clinical response in UC patients. Patients who received 100 mg golimumab every 4 weeks had higher clinical remission and mucosal healing rates (27.8% and 42.4%) than patients receiving placebo (15.6% and 26.6%; P = 0.004 and P = 0.002, respectively) | Infections, nasopharyngitis, abdominal pain, rash; pharyngitis, cough | (74) |
| A retrospective study | CD patients | Golimumab | 9.8 months (0.55-44 months) | 18.2 months (2.5-44.0 months) | 55.8% of the patients achieved clinical response after a mean duration of 3.8 months. 6% of the patients discontinued treatment due to intolerance | NA | (76) |
| A single center, retrospective study | Patients with refractory UC | IFX intensification (a dose escalation (up to 10 mg/kg) and/or shorter intervals between infusions (every 4-6 weeks)) | NA | NA | Clinical remission was observed in 72.7% of the patients within 8 weeks after initiating IFX treatment. IFX intensification was necessary for long-term maintenance of remission and to prevent colectomy in patients with refractory UC | Infusion reactions | (78) |
| A randomized, double-blind trial | Anti-TNF-α-naive adults with moderate to severe UC | Combination therapy with IFX (5 mg/kg at weeks 0, 2, 6, and 14) and AZA (2.5 mg/kg daily) | 16 weeks | 16 weeks | Combination therapy with IFX and AZA achieved higher corticosteroid-free remission (39.7%) and mucosal healing (62.8%) rates, compared with patients receiving IFX (P = 0.017) or AZA (P = 0.032) alone | Headache, nausea, vomiting, pyrexia, hepatobiliary events, anemia,  fatigue | (79) |
| A multicenter, randomized, prospective, open-labelled study | Patients with moderately to severely active CD | Combination therapy with ADA and AZA or monotherapy with ADA | 52 weeks | 52 weeks | The combination therapy with ADA and AZA may be more beneficial for CD patients compared with ADA monotherapy | NA | (81) |
| Three randomized controlled phase 3 studies | Patients with moderate to severe CD | Intravenous ustekinumab (130 mg or 6 mg/kg) for induction therapy; subcutaneous ustekinumab (90 mg every 12 weeks or 8 weeks) for maintenance therapy | 8 weeks; 8 weeks; 44 weeks | 8 weeks; 8 weeks; 44 weeks | Ustekinumab was effective for inducing and maintaining endoscopic healing in patients with moderate to severe CD | NA | (85) |
| Three double-blind, placebo-controlled trials | Patients with moderately to severely active CD | Intravenous ustekinumab (130 mg or 6 mg/kg) for induction therapy; subcutaneous maintenance injections of 90 mg of ustekinumab (every 8 weeks or every 12 weeks) | 6 weeks; 6 weeks; 44 weeks | 6 weeks; 6 weeks; 44 weeks | Rates of clinical response at week 6 among patients receiving intravenous ustekinumab at a dose of 130 mg or 6 mg/kg were significantly higher than the rates among patients receiving placebo (in UNITI-1, 34.3%, 33.7%, and 21.5%, respectively, with P≤0.003 for both comparisons with placebo; in UNITI-2, 51.7%, 55.5%, and 28.7%, respectively, with P<0.001 for both doses). In the groups receiving maintenance doses of ustekinumab every 8 weeks or every 12 weeks, respectively, were in remission at week 44, as compared with 35.9% of those receiving placebo (P=0.005 and P=0.04, respectively). Clinical remission at week 44 was observed in 53.1% of patients receiving maintenance doses of ustekinumab every 8 weeks and 48.8% receiving ustekinumab every 12 weeks, compared with 35.9% of those receiving placebo (P<0.05, for both groups) | Arthralgia, headache, nausea, pyrexia, nasopharyngitis, abdominal pain, fatigue, infections | (86) |
| A phase 2, randomized trial | Patients with moderately to severely active UC | Intravenous mirikizumab (50 mg or 200 mg with exposure-based dosing, or 600 mg with fixed dosing at weeks 0, 4, and 8) for induction therapy; subcutaneous mirikizumab (200 mg every 4 or every 12 weeks) | NA | 52 weeks | 22.6% of patients in the 200-mg group achieved clinical remission, compared with 4.8% of patients given placebo (P = 0.004) at week 12. At week 52, clinical remission was observed in 46.8% of patients given subcutaneous mirikizumab 200 mg every 4 weeks and 37.0% given subcutaneous mirikizumab 200 mg every 12 weeks | Nasopharyngitis, anaemia, headache, nausea, cough, upper respiratory tract infection, arthralgia, hypertension, influenza during maintenance | (87) |
| A randomized, double-blind, placebo-controlled phase 2 study | Patients with moderately-to-severely active CD | Risankizumab (BI 655066, Boehringer Ingelheim, Ingelheim, Germany) (intravenously, 200 or 600 mg at weeks 0, 4, and 8) | 8 weeks | 12 weeks | Risankizumab was more effective for inducing clinical remission compared with placebo. Rate of clinical remission in patients given 600 mg risankizumab was higher than patients receiving placebo (37% vs. 15%, p<0.05) | Nausea, abdominal pain, arthralgia, anaemia, headache, vomiting | (88) |
| An open-label extension study of a randomized, phase 2 induction study | Patients with moderately to severely active CD who completed the 12-week induction phase of the double-blind phase 2 study | Risankizumab (intravenously, 200 or 600 mg at week  0, 4, and 8); (intravenously, 600 mg every 4 weeks for 12 weeks); (subcutaneously, 180 mg every 8 weeks for 26 weeks) | 12 weeks; 12 weeks; 26 weeks | 52 weeks | Extended induction treatment with open-label intravenous risankizumab was effective in increasing clinical response and remission rates at 26 weeks. Open-label subcutaneous risankizumab maintained remission until week 52 in most patients who were in clinical remission at week 26 (Rates of clinical remission, clinical response, endoscopic remission, endoscopic response, mucosal healing and deep remission were 71%, 81%, 35%, 55%, 24% and 29%, respectively) | Arthralgia, headache, abdominal pain, nasopharyngitis, nausea, pyrexia | (89) |
| Two integrated randomized, double-blind, placebo-controlled trials | Patients with active UC | Intravenous vedolizumab 300 mg at weeks 0 and 2 for induction therapy; vedolizumab every 8 or 4 weeks for maintenance therapy | 52 weeks | 52 weeks | Vedolizumab was more effective than placebo as induction and maintenance therapy for UC. Patients in the vedolizumab group achieved a higher rate of response at week 6 than patients in placebo group (47.1% vs. 25.5%, P<0.001). At week 52, 41.8% of patients who continued to receive vedolizumab every 8 weeks and 44.8% of patients who continued to receive vedolizumab every 4 weeks were in clinical remission, as compared with 15.9% of patients who switched to placebo (P<0.001, for both comparisons with placebo) | Headache, nasopharyngitis,  upper respiratory tract, infection, abdominal pain, fatigue, cough | (94) |
| A phase 3, randomized, parallel-group, double-blind, placebo-controlled study | Adults with active CD | Intravenous vedolizumab (300 mg) | 52 weeks | 52 weeks | Vedolizumab was more effective for inducing and maintaining clinical remission than placebo but associated with a higher rate of adverse events. At week 6, 14.5% of the patients who received vedolizumab and 6.8% who received placebo were in clinical remission (P=0.02); a total of 31.4% and 25.7% of the patients, respectively, had a clinical response (P=0.23). Among patients who had a response to induction therapy, 39.0% and 36.4% of those assigned to vedolizumab every 8 weeks and every 4 weeks, respectively, were in clinical remission at week 52, as compared with 21.6% assigned to placebo (P<0.001 and P=0.004 for the two vedolizumab groups, respectively, vs. placebo) | Nasopharyngitis, arthralgia, nausea, upper respiratory tract infection, fatigue, back pain, serious infections | (95) |
| Six clinical studies | Patients with UC or CD | Vedolizumab | NA | NA | Vedolizumab was well-tolerant with low incidence rates of serious infections, infusion-related reactions and malignancies over a long-term treatment | Nasopharyngitis, abdominal pain, headache, arthralgia | (96) |
| A long-term follow-up study | Patients with UC or CD | Vedolizumab along with calcineurin inhibitors | 12 months | 52 weeks | Combination therapy of vedolizumab with either CsA or TAC was effective and safe at inducing and maintaining clinical remission in patients with UC or CD. By week 14 of treatment, steroid-free clinical remission was achieved in 44% of the patients with CD and 55% of the patients with UC; 33% of the patients with CD and 45% of the patients with UC were in steroid-free clinical remission at week 52 | Infections, pruritis, rheumatologic, paresthesia, migraine, mild tremor, constipation, perianal disease, fatigue | (98) |
| A retrospective observational study | Patients with an active steroid-refractory UC | calcineurin inhibitors for induction therapy and vedolizumab for maintenance | NA | 10.8 months (0.4-30.2 months) | Combining calcineurin inhibitors  with vedolizumab in patients with steroid-refractory UC and prior anti-TNF failure or contraindication can avoid 1-year colectomy in about two thirds of the patients | Kidney failure, campylobacter colitis, headache, gingivitis, dry syndrome, asthenia, edema | (99) |
| A phase 2, randomized, placebo-controlled trial | Patients with moderate to severe UC who had not responded to conventional therapy | subcutaneous etrolizumab (100 mg at weeks 0, 4, and 8, with placebo at week 2; or 420 mg loading dose at week 0 followed by 300 mg at weeks 2, 4, and 8) | 8 weeks | 10 weeks | Patients in the etrolizumab 100 mg group had higher clinical remission rates than patients receiving placebo at week 10 (21% vs. 0%, p=0.0040) | Rash, influenza-like illness, arthralgias | (100) |
| A double-blind, placebo-controlled, phase 2a study | Patients with moderately active UC who had inadequate response or intolerance to mesalamine or CSs | AJM300 (960 mg 3 times daily) | 8 weeks | 8 weeks | AJM300 was well tolerated and resulted in higher rates of clinical response (62.7% vs. 25.5%, P=0.0002), clinical remission (23.5% vs. 3.9%, P=0.0099), and mucosal healing (58.8% vs. 29.4%, P=0.0014) than placebo | Nasopharyngitis, headache, nausea, blood amylase increased, blood lactate dehydrogenase increased, white blood cell count increased | (102) |
| A phase 2, randomized, double-blind, placebo-controlled trial | Patients with moderate to severe UC who had failed or were intolerant to at least one conventional therapy | Anti-MAdCAM antibody (PF-00547659) (subcutaneously, 7.5 mg, 22.5 mg, 75 mg, or 225 mg every 4 weeks) | 12 weeks | 12 weeks | PF-00547659 appeared safe and effective for UC patients in inducing remission. Remission rates at week 12 were significantly greater in 22.5 mg- PF-00547659 group (16.7%) and 75 mg group (15.5%) than in the placebo group (2.7%) | Abdominal pain, nausea, vomiting, headache | (103) |

*UC, ulcerative colitis; IFX, Infliximab; CD, Crohn’s disease; NA, not applicable; ADA, Adalimumab; AZA, azathioprine; CsA, Cyclosporine A; TAC, Tacrolimus; TNF, tumor necrosis factor; MAdCAM, mucosal addressin cell adhesion molecule.*
